# Effects of different treatment measures on the efficacy of diabetic foot ulcers: a network meta-analysis

**DOI:** 10.3389/fendo.2024.1452192

**Published:** 2024-09-23

**Authors:** Hong OuYang, Jing Yang, Haiyan Wan, Jiali Huang, Yifan Yin

**Affiliations:** ^1^ Geriatric Diseases Institute of Chengdu, Department of Endocrine and Metabolism, Chengdu Fifth People’s Hospital(The Second Clinical Medical College, Affiliated Fifth People’s Hospital of Chengdu University of Traditional Chinese Medicine), Chengdu, China; ^2^ Department of Nephrology, Chengdu Third People’s hospital, Chengdu, China

**Keywords:** diabetic foot ulcers, ultrasonic debridement, negative pressure wound therapy, stem cells, hyperbaric oxygen therapy, topical oxygen therapy, platelet-rich plasma, acellular dermal matrix

## Abstract

**Introduction:**

Through a network meta-analysis, we compared different treatment measures for patients with diabetic foot ulcers (DFU), assessing their impact on the healing of DFU and ranking them accordingly.

**Methods:**

We searched the PubMed, the China National Knowledge Infrastructure (CNKI), Embase, the WanFang and the WeiPu database. The retrieval time was from database establishment to January 2024, and retrieval entailed subject and free words. Randomized controlled trials (RCTs) with different treatment measures for DFU were included. Data extraction and evaluation were based on the PRISMA guidelines. Meta-analyses using pairwise and network methods were employed to compare and rank the effectiveness of different treatments for DFU.

**Results:**

Ultimately, we included 57 RCTs involving a total of 4,826 patients with DFU. When it comes to ulcer healing rates, compared to standard of care(SOC),platelet-rich plasma(PRP), hyperbaric oxygen therapy(HBOT), topical oxygen therapy(TOT), acellular dermal matrix(ADM), and stem cells(SCs) in both direct meta-analysis(DMA) and network meta-analysis(NMA) can effectively increase the complete healing rate. For Scs+PRP, a statistically significant improvement was only observed in the NMA. Moreover, when compared to the negative pressure wound therapy(NPWT) group, the PRP+NPWT group was more effective in promoting the complete healing of ulcers. In terms of promoting the reduction of ulcer area, no statistical differences were observed among various treatment measures. When it comes to ulcer healing time, both PRP and NPWT can effectively shorten the healing time compared to SOC. Furthermore, when compared to the NPWT group, the combined treatment of PRP and ultrasonic debridement(UD) with NPWT is more effective in reducing healing time. In terms of amputation rates and adverse reactions, the PRP group effectively reduced the amputation rate and adverse reactions for patients with DFU. Additionally, compared to the NPWT group, the combined treatment of PRP and UD with NPWT reduced the incidence of adverse reactions. However, no significant differences were observed among other treatment measures in terms of amputation rates and adverse reactions. The ranking results showed that the efficacy of PRP+NPWT and UD+NPWT in promoting ulcer healing, reducing ulcer area, shortening healing time, decreasing amputation rates and adverse reactions is superior to that of the alone PRP group, NPWT group, and UD group. Conversely, the SOC group demonstrates the least effective performance in all aspects.

**Conclusion:**

Due to the particularity of the wound of DFU, the standard of care is not effective, but the new treatment scheme has a remarkable effect in many aspects. And the treatment of DFU is not a single choice, combined with a variety of methods often achieve better efficacy, and will not bring more adverse reactions.

## Introduction

Diabetes (DM) is a rapidly spreading disease worldwide, posing a significant health challenge globally (1). It is estimated that there were 451 million patients with diabetes in 2017, and this number is projected to rise to 693 million by 2045 (2). Diabetic foot ulcer (DFU) is one of the clinical manifestations of diabetic nerve lesions, defined as structural or functional changes in the foot associated with diabetic nerve lesions and varying degrees of peripheral vascular disease, such as ulcers, infections, or gangrene (3). DFU is one of the most common, most severe, and most costly complications of diabetes (4–7), with approximately 19% to 34% of patients with diabetes experiencing a DFU in their lifetime (8). It is characterized by complex management, high incidence rate, and high mortality rate (9). The global incidence rate of diabetes foot ulcers is approximately 6.3%, occurring predominantly in patients with type 2 diabetes(T2DM), the elderly, and those with a prolonged history of diabetes (10). According to predictions from the World Health Organization (WHO), by 2030, DFU will affect more than 19% of the world’s adult population (11). DFU are also the primary reason for hospitalizations among diabetes patients worldwide. Reports indicate that nearly 88% of lower leg amputations are associated with DFU, often resulting in disability and severely compromising the quality of life (12, 13). The 5-year mortality rate for patients with DFU is 30%, while the 5-year mortality rate for those undergoing amputations exceeds 70% (14). Furthermore, the annual cost associated with DFU treatment and amputations is extremely high, approximately 10.9 billion USD worldwide (15). From this, it is evident that DFU are associated with significant incidence rates and mortality rates, as well as imposing a substantial economic, social, and public health burden.

Currently, the standard treatments for DFU includes debridement, dressing, offloading, vascular assessment, infection management, and blood glucose control

(16). However, these treatments are not satisfactory. It has been reported that the complete healing rates for DFU patients after 12 weeks and 20 weeks of standard therapy are only 24% and 31% respectively (17). Therefore, in recent years, several adjunctive techniques have been developed for the debridement treatment of DFU. These include ultrasonic debridement (UD), negative pressure wound therapy (NPWT) [including vacuum-assisted closure (UAC) and vacuum sealing drainage (VSD)], and oxygen therapies [such as hyperbaric oxygen therapy (HBOT) and topical oxygen therapy (TOT)]. Additionally, studies have found that using stem cells (SCs), growth factors, or tissue-engineering dressings can form the basis for a new treatment approach. Among these, fat-derived SCs, platelet-rich plasma(PRP), and acellular dermal matrix(ADM) have emerged as focal points of research. These treatment methods aim to restore the body’s natural healing process (18). Direct meta-analysis(DMA) have been employed to compare the efficacy and safety of these therapeutic measures in the treatment of DFU (19–26), yet divergent opinions persist, such as:Tasmania et al. concluded that the use of PRP in DFU promoted wound healing, reduced ulcer volume, reduced the time to complete wound healing, and reduced the incidence of adverse events, with no difference in the probability of wound complications (27). This is consistent with previous findings (23, 25, 28). However, Ajay et al. concluded that PRP had no significant effect on promoting ulcer healing (29); Zhao et al. found no difference in ulcer incidence, risk of amputation, or adverse events with HBOT compared to standard treatment (ST) (30). Sharma et al. believe that HBOT has significant effect on the complete healing of diabetic foot ulcers, and can shorten the healing time and reduce the incidence of major amputations (24). Moreover, there have been no studies that directly compared the therapeutic outcomes of these varied treatments for DFU. In contrast, network meta-analysis(NMA) can utilize both direct and indirect data to compare various interventions, and by ranking the therapeutic effects of all interventions, they can identify the most effective treatment method. Consequently, to further evaluate the impact of different therapeutic methods on the outcome of DFU efficacy, we included relevant randomized controlled trials (RCTs) in a NMA, aiming to provide stronger evidence for the effectiveness and safety of various treatments for DFU.

## Materials and methods

Our study follows the recommendations of the assessing the methodological quality of systematic reviews(AMSTAR)guidelines (31) and is consistent with the preferred reporting items for systematic reviews and meta-analyses(PRISMA)statement (32).

### Search strategy

We searched the PubMed, the China National Knowledge Infrastructure (CNKI), Embase, the WanFang and the WeiPu database. The retrieval time was from database establishment to January 2024, and retrieval entailed subject and free words. The search terms were included in the abstract or title, include “diabetic foot ulcers,” “platelet-rich plasma,” “negative pressure wound therapy,” “hyperbaric oxygen therapy,” “topical oxygen therapy,” “ultrasonic debridement,” “acellular dermal matrix,” “stem cells,” and “randomized controlled trials.” The publication type of the studies is restricted to randomized controlled trials (without language or location limitations).

### Inclusion and exclusion criteria

Studies included in the NMA must meet the following criteria: (1) The subjects are patients with DFU, regardless of age, gender, race or nationality. (2) The study type is a RCT. (3) The study divided the participants into two or more groups, and patients in each group were treated with one treatment or a combination of treatment measures. The therapeutic measures include: platelet-rich plasma, negative pressure wound therapy, hyperbaric oxygen therapy, topical oxygen therapy, ultrasonic debridement, acellular dermal matrix, stem cell transplantation and standard treatments, so as to compares the efficacy and safety of different treatment measures in patients with DFU. (4) The study provides at least one effective efficacy indicator: complete healing rate, healing time required, reduction in ulcer area, amputation rate, and adverse reactions (infection, allergy, pain, etc.).

Exclusion criteria: (1) Studies that do not meet the criteria for RCTs, including: reviews, commentaries, letters, etc. (2) Exclusion of studies that did not include relevant outcome measures (3) Exclusion of subjects who do not meet the diagnostic criteria for DFU. (4) Repeated publications. (5) Documents with incomplete or insufficient data.

### Outcome indicator

The primary outcome measures in the NMA studies mainly include ulcer healing rate, time required for ulcer healing, reduction in ulcer area, amputation rate, and adverse reactions (infection, allergy, pain, etc.).

### Data extraction and quality evaluation

Two evaluators independently searched the database based on inclusion and exclusion criteria, searching the full text of the initially included articles. They used a uniform form to extract data, including: author name, publication year, country, research subjects (sample size, gender ratio, average age, and smoking history), interventions (experimental group and control group), duration of the study, duration and area of DFU, and main research results. The methodological quality of the study was evaluated in accordance with the Cochrane Risk Bias tool. Evaluated aspects included the following: random sequence generation, hidden distribution concealment, the blinding of subjects and intervention providers, the blinding of result evaluation, the integrity of the outcome data, selective result reporting, and other sources of bias. When there was inconsistency, judgment was reached through public discussion.

### Statistical analysis

#### Direct meta-analysis

For DMA, we used STATA12.0 software for statistical analysis, using odds ratio (OR) and 95% confidence interval (CI) as the evaluation index of the results, represented by mean difference and 95% CI. First, heterogeneity was assessed using the X^2 test (a=0.05) and a quantitative analysis of I^2 for heterogeneity (I^2 ≥ 50%) conducted. In cases of no heterogeneity between the research results, the meta-analysis was conducted. In cases of statistical heterogeneity between the research results, the source of heterogeneity was further analyzed, and the random heterogeneity model was used after excluding the influence of obvious clinical heterogeneity. Funnel maps created using the STATA software were employed to detect publication bias.

#### Network meta-analysis

We performed a Bayesian NMA using R and STATA software. NMA can combine direct and indirect comparisons to further analyze the effects of different treatment options on DFU. The results of the comparison effect are expressed as OR and its 95% CI. Moreover, we built a network diagram using the mtc.network”command of the gemtc”package in the R software. Furthermore, we calculated the percentage area under the cumulative ranking (SUCRA) curve, ranking the different interventions. One intervention had a higher SUCRA value than others, indicating that the better the treatment effect, the lower the incidence of adverse reactions. A node splitting method was used to evaluate the consistency hypothesis of direct and circumstantial evidence. When direct evidence of the results was consistent with circumstantial evidence (P> 0.05), the consistency model was adopted.

## Results

### Retrieved results

**Figure 1:**
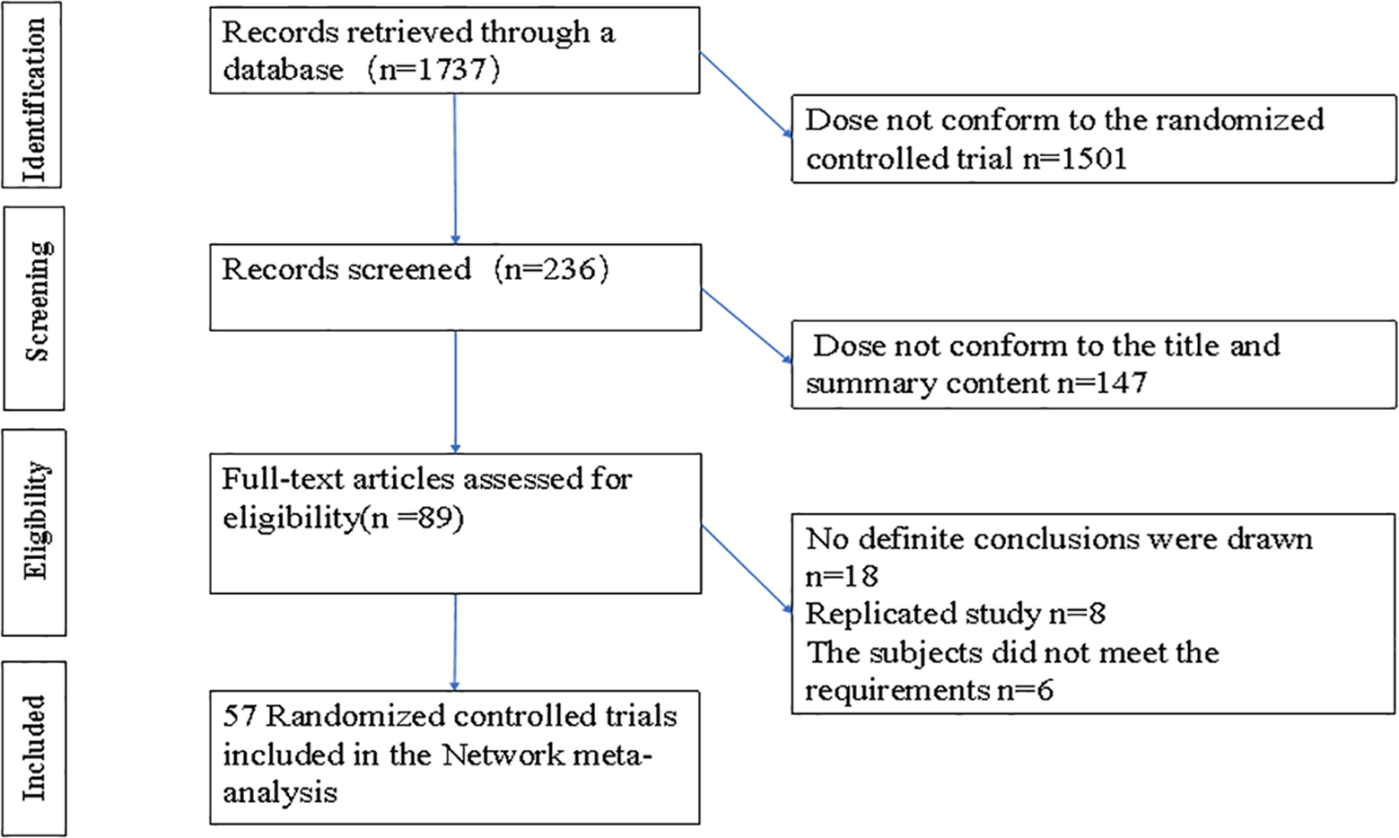
PRISMA flow diagram for the literature search and study selection.

Based on a pre-designed literature search strategy, a total of 1,737 articles were identified, of which 1,501 were not classified as RCTs. After reviewing the titles and abstracts, 147 articles were excluded, allowing for a detailed review of 89 articles. Ultimately, we included 57 RCTs involving a total of 4,826 patients with DFU. Other studies were excluded: 18 did not have clear efficacy outcome indicators, 8 were repetitive studies, and 6 did not meet the criteria for diabetes foot. The literature screening process and results are shown in **Figure 1**.

### Characteristics and quality of research

We have summarized the fundamental characteristics of the included studies, as shown in **Table 1**. The studies were published between 2009 and 2024, with 34 originating from Asia (29, 38, 40, 42, 45, 48–50, 57–63, 65–74, 76, 77, 81–87), 11 from North America (33, 35–37, 39, 51–56), 6 from Europe (34, 41, 43, 46, 47, 64), and 6 from Africa (44, 75, 78–80). The included studies encompassed 11 distinct interventions: PRP, NPWT (including UAC and VSD), HBOT, TOT, UD,ADM, SCs, SOC, PRP+SCs, PRP+NPWT, and NPWT +UD. Among them, there are 15 studies comparing PRP vs SOC, 6 studies on NPWT vs SOC, 7 studies on HBOT vs SOC, 5 studies on TOT vs SOC, 2 studies on UD vs SOC, 6 studies on ADM vs SOC, 3 studies on SCs vs SOC, 1 study on PRP vs HBOT vs SOC, 3 studies on PRP+NPWT vs NPWT, 2 studies on PRP+NPWT vs SOC, 5 studies on NPWT+UD vs NPWT, and 2 studies on SCs vs PRP +SCs vs SOC.

**Table 1:** Characteristics of studies included in the network meta-analysis.

Author,published year and country	Groups (n)	Study period (weeks)	Mean age (years)	Male gender (n)	Wound duration (weeks)	Wound area (cm2)	Primary outcomes
Driver,2017,UAS (33)	SOC(61)/TOT(61)	12	58.8 ± 9.4/58.6 ± 12.31	47/43	14.9 ± 12.5/17.7 ± 12.8	2.3 ± 1.7/2.0 ± 1.7	Complete wound healing
Frykberg,2020,UK (34)	SOC(36)/TOT(37)	12	61.9 ± 9.5/64.6 ± 10.3	31/32	24.9 ± 13.4/22.9 ± 13.7	3.22 ± 2.54/3.02 ± 2.66	Complete wound healing
Niederauer,2017,UAS (35)	SOC(50)/TOT(50)	12	59.1 ± 13.3/57.5± 10.9	40/39	NA	3.9 ± 2.0/3.4 ± 1.5	Rates of closure, and time to closure
Niederauer,2018,UAS (36)	SOC(72)/TOT(74)	12	56.6 ± 14.4/56.1 ± 10.1	54/59	20.54 ± 13.96/18.8 ± 12.7	3.89 ± 2.02/3.54 ± 1.68	Full wound closure
Janelle,2016,Canada (37)	SOC(10)/TOT(10)	8	58± 9.5/57 ± 9.5	NA	46.2 ± 17.9/47.4 ± 23.4	1.68 ± 1.31/1.37 ± 0.95	Rates of closure, and time to closure
Chen,2017,China (38)	SOC(18)/HBOT(20)	4	60.8 ± 7.2/64.3 ± 13.0	11/10	4.98 ± 4.8/8.44 ± 6.97	NA	Physiological indices and blood biochemistry tests
Fedorko,2016,Canada (39)	SOC(54)/HBOT(49)	12	62/61	38/31	48/33	5.1/6.1	Amputation and wound healing
Ma,2013,China (40)	SOC(18)/HBOT(18)	2	60.4 ± 5.6/59.8 ± 6.5	12/11	45.2 ± 34/57.2 ± 46.4	4.35 ± 1.04/4.21 ± 0.99	Complete wound healing
Santema,2018, Netherlands (41)	SOC(60)/HBOT(60)	48	70.6 ± 11.2/67.6 ± 10.0	46/51	24/22.4	3.5 ± 2.9/3.2 ± 2.7	Limb salvage and wound healing after 12 months, as well as time to wound healing.
Chaudhary,2013,India (42)	SOC(20)/HBOT(20)/PRP(20	60	45 ± 7.5/43.8 ± 9.4/43.3 ± 8.1	11/10/11	6.75 ± 2.65/6.83 ± 2.5/7.6 ± 2.53	9.9 ± 5.5/14.9 ± 6.2/19.2 ± 11.3	Complete wound healing
Londahl,2010, Sweden (43)	SOC(45)/HBOT(49)	8	69/68	38/38	40/36	2.8/3.1	Complete wound healing
Salama,2019,Egypt (44)	SOC(15)/HBOT(15)	8	57.7 ± 6.7/55.1 ± 7.5	10/12	15.5 ± 1.4/16.5± 1.5	8(2-16.5)/7.5 (1.5-15.5)	Complete healing of the target ulcer
Kumar,2020,India (45)	SOC(26)/HBOT(28)	6	56.9 ± 11.1/58.4 ± 10.1	19/20	36 ± 11.6/32 ± 8.4	3.0 ± 2.8/2.9 ± 1.5	Healing and need for amputation,grafting or debridement
Lonardi,2019,Italy (46)	SOC(50)/SCs(55)	24	71.6 ± 10.8/69.0 ± 11.6	41/45	NA	NA	The healing rate and time
Smith,2020, UK (47)	SOC(6)/SCs(6)/PRP+SCs(6)	12	55.2/60.2/57.5	4/6/5	49/41/54	0.64/0.31/0.13	Wound size and Wound healing
E.Uzun,2020. Turkey (48)	SOC(10)/SCs(10)	24	57.2 ± 4.5/57.5 ± 8.4	6/6	6.8 ± 2.4/7.8 ± 2.9	25.8 ± 5.4/23.5 ± 5.6	Wound characteristics, wound closure time, amputation rates and clinical scores
Han,2010,Korea (49)	SOC(26)/SCs(26)	8	68.4 ± 8.7/66.5 ± 7.5	14/15	4.0 ± 2.1/4.3 ± 2.1	12.5 ± 5.5/12.5 ± 5.6	The percentages of complete healing and mean healing times
Meamar,2021,Iran (50)	SOC(7)/SCs(11)/SCs+PRP(10)	16	56 ± 10.5/68 ± 8.1	NA	NA	8.6 ± 5.5/11.11 ± 5.8,/11.2 ± 5.6	Wound area and pain free walking distance
Brigido,2006,USA (51)	SOC(14)/ADM(14)	16	66/61	NA	NA	NA	Complete wound healing
Driver,2015,USA (52)	SOC(153)/ADM(154)	16	57.3 ± 9.7/55.8 ± 10.6	114/118	NA	3.65 ± 2.7/3.53± 2.5	Complete wound healing
Walters,2016,USA (53)	SOC(56)/ADM(76)	16	57.1 ± 10.9/58.4 ± 11.7	NA	NA	3.3 ± 2.5/3.6 ± 4.2	Complete wound healing
Reyzelman,2009, USA (54)	SOC(39)/ADM(46)	12	58.9 ± 11.6/55.4 ± 9.6	NA	22.9 ± 12.0/23.3 ± 16.0	5.1 ± 3.2/3.6 ± 2.2	The proportion of healed diabetic foot ulcers and mean healing time
Zelen, 2016, USA (55)	SOC(20)/ADM(20)	12	57.1 ± 10.65/61.5 ± 10.85	12/16	NA	2.7 ± 2.26/4.7 ± 5.24	Complete wound healing
Zelen, 2018, USA (56)	SOC(40)/ADM(40)	12	62/59	24/28	NA	2.7/3.2	The proportion of wounds closed
Gao, 2022, China (57)	SOC(50)/UD(50)	4	66/67	26/27	NA	14.5 ± 0.9/15.6 ± 0.8	Patients total clinical efficiency, adverse events, ulcer areas, healing rate, and positive bacterial culture rate
Rastogi,2019,India (58)	SOC(50)/UD(50)	4	51.2 ± 7.3/52.5 ± 7.2	NA	12.1 ± 10.9/15.8 ± 11.2	14.8 ± 13.8/11.3± 8.2	A >50% reduction in wound area
Li,2016,China (59)	NPWT(35)/NPWT+UD(35)	NA	40.13 ± 13.2/40.43 ± 12.0	23/21	25.3 ± 12.4/23.3 ± 11.3	30 ± 23.4/36 ± 19.8	The ulcer area,granulation tissue coverage rate and ulcer recurrence rate
Wang,2018,China (60)	NPWT(30)/NPWT+UD(30)	NA	61.5 ± 8.5/60.5 ± 8.5	20/18	NA	1.81 ± 0.42/4.04 ± 0.76	The number of changing times, the wound healing time and the incidence of adverse reactions
Li,2022,China (61)	NPWT(20)/NPWT+UD(20)	NA	61.0 ± 9.81/58.85 ± 6.98	15/13	NA	NA	The treatment effects,pain degree, ankle-brachial index and complication rate
Diao,2022,China (62)	NPWT(30)/NPWT+UD(30)	2	49.85 ± 2.26/49.97 ± 2.39	17/19	18.3 ± 4.6/18.5 ± 4.5	12.42 ± 1.18/12.19 ± 1.21	The clinical treatment effect, pain score and expression of serum inflammatory factors
Ji,2019,China (63)	NPWT(76)/NPTW+UD(77)	NA	56.12 ± 8.60/55.75 ± 8.51	38/42	4.7 ± 0.7/32.4.7 ± 0.6	19.28 ± 6.42/15.28 ± 5.79	The bacterial clearance rate, wound surface reduction and wound surface reduction rate
Seidel,2020,Germany (64)	SOC(174)/NPWT(171)	16	68.1/67.6	134/133	23.2 ± 12.1/31.0 ± 11.6	11.4 ± 4.7/10.6± 5.5	Wound closure
Maranna,2021,India (65)	SOC(23)/NPWT(22)	21	49.00 ± 10.14/50.23 ± 10.52	17/16	38.9 ± 12.9/36.9 ± 12.5	47.30 ± 17.00/48.45 ± 17.42	The formation of granulation tissue, reduction in ulcer size, duration of hospital stay and time for complete healing of wounds
Vivek,2022, India (66)	SOC(32)/NPWT(23)	3	36.74 ± 7.22/37.32 ± 6.84	26/16	30.2 ± 10.9/34.2 ± 8.5	19.87 ± 3.66/20.46 ± 3.45	Wound healing
Sangma,2019,India (67)	SOC(32)/NPWT(23	3	52.89/55.85	15/16	NA	80.44/70.97	The time to wound healing, granulation tissue formation, and complications such as pain, infection, and bleeding
Erhan,2018,Turkey (68)	SOC(34)/NPWT(31)	NA	58.3 ± 8.0/60.6 ± 11.6	27/25	20.3 ± 11.2/25.2 ± 14.7	17.6 ± 3.3/18.3 ± 3.1	Wound healing
Muhammad,2015, Pakistan (69)	SOC(139)/NPWT(139)	2	55.88 ± 10.97/56.83 ± 11.3	114/107	NA	15.07 ± 2.92/15.09 ± 2.81	Reduction in wound area
Chen,2021, China (70)	NPWT(50)/PRP+ NPWT(50)	6	60.88 ± 7.98/61.62 ± 8.35	27/29	NA	NA	The total effective rates and the effect of wound repair
Gao, 2021, China (71)	NPWT(49)/NPWT+PRP(49)	NA	59. 2± 6. 7/60. 7 ± 6. 9	29/30	NA	NA	The formation of granulation tissue and new blood vessels, healing time
Chen,2018, China (72)	SOC(30)/PRP+ NPWT(28)	NA	49.59 ± 1.54/49.54 ± 1.55	12/13	NA	NA	The hospitalization time and ulcer healing time
Niu,2020,China (73)	SOC(35)/PRP+NPWT(38)	NA	59.62 ± 5.33/58.49 ± 4.97	19/20	NA	NA	Wound healing
Wan, 2021, China (74)	NPWT(55)/PRP+NPWT(55)	2	54.66 ± 8.79/53.25 ± 9.56	27/28	NA	2.49 ± 0.26/2.57 ± 0.52	Treatment effect, ulcer healing time,hospital stay,blood biochemical indexes before and after treatment
Ahmed,2016,Egypt (75)	SOC(28)/PRP(28)	12	49.8 ± 15.4/43.2± 18.2	18/20	11.5 ± 2.8/12.5 ± 1.0	5.72 ± 0.48/6.24 ± 0.77	Complete healing
Ajay,2021,India (29)	SOC(30)/PRP(30)	6	55.76 ± 10.2/56.03± 9.6	19/22	44.9 ± 70.8/54.8 ± 70.3	4.96± 2.89/5.22 ± 3.82	Wound healing
Alamdari,2021,Iran (76)	SOC(47)/PRP(43)	24	56.7 ± 7.2/56.3 ± 7.1	30/26	NA	NA	The healing rate of foot ulcers
Shailendra,2018,India (77)	SOC(26)/PRP(29)	4	55.69 ± 10.35/53.76 ± 10.38	15/19	NA	NA	Wound healing
Ahmed,2019,Egypt (78)	SOC(12)/PRP(12)	20	55.6 ± 6.5/54.7 ± 6.6	6/8	22.3± 10.8/21 ± 13.6	15.16 ± 2.1/25.58± 8.32	Percent reduction in the dimensions of the DFU, healing of DFU, and complications at 20 weeks of follow-up
Yasser,2022,Egypt (79)	SOC(36)/PRP(36)	20	58.69 ± 6.68/56.03± 8.39	21/20	46.67 ± 39.87/46.64 ± 31.47	3.22 ± 1.2/3.33± 1.31	Wound healing
ElMahdy,2021,Egypt (80)	SOC(40)/PRP(40)	12	54.8± 3.9/54.9 ± 2.37	34/28	NA	14.5 ± 5.6/15.2 ± 5.6	Wound healing and decrease the rate of local infection
Xie,2019,China (81)	SOC(23)/PRP(25)	8	61.10 ± 7.90/60.50 ± 8.27	13/14	24.3 ± 16.96/21.60 ± 18.50	11.78 ± 7.78/11.84 ± 9.67	The sinus tract closure times, ulcer healing rates, hospitalization times, and hospitalization expenses
Li,2014,China (82)	SOC(55)/PRP(48)	12	64.1 ± 9.4/61.4 ± 13.1	38/37	3.3/4.3	2.9/4.1	Wound healing
Li,2022,China (83)	SOC(36)/PRP(36)	12	64.2 ± 9.8/62.5 ± 10.1	20/22	3.1 ± 2.4/3.3 ± 2.1	26.9 ± 18.3/28.2 ± 17.8	Healing time (days), length of hospital stay (days), healing rate, surface area reduction (cm2), and adverse events
Asad,2022,Pakistan (84)	SOC(80)/PRP(80)	24	57.7 ± 10.1/54.4 ± 8.5	14/13	NA	NA	Wound healing
Du,2022,China (85)	SOC(30)/PRP(30)	14	53.4/54.44	17/19	NA	7.244 ± 1.24/7.4 ± 1.6	Healing area, volume, and rates
Jeong,2010,Korea (86)	SOC(48)/PRP(52)	12	63.8 ± 6.4/64.5± 8.1	26/27	10.1± 3.1/12.4 ± 5.6	5.3 ± 2.2/5.7 ± 3.6	The percentage of complete healing, mean healing time, percentage of wound shrinkage, and patient satisfaction
Hossam,2021,Egypt (80)	SOC(40)/PRP(40)	12	54.8 ± 3.9/54.9± 2.37	34/28	NA	14.5 ± 5.6/15.2 ± 5.6	Wound healing and the rate of local infection
Arash,2022,Iran (87)	SOC(81)/PRP(81)	12	60.2 ± 5.2/55.8 ± 5.6	46/52	6.4 ± 1.8/6.0 ± 0.7	3.3 ± 0.5/3.2 ± 0.5	The percentage area reduction

NA, Not Available.

**Figure 2:**
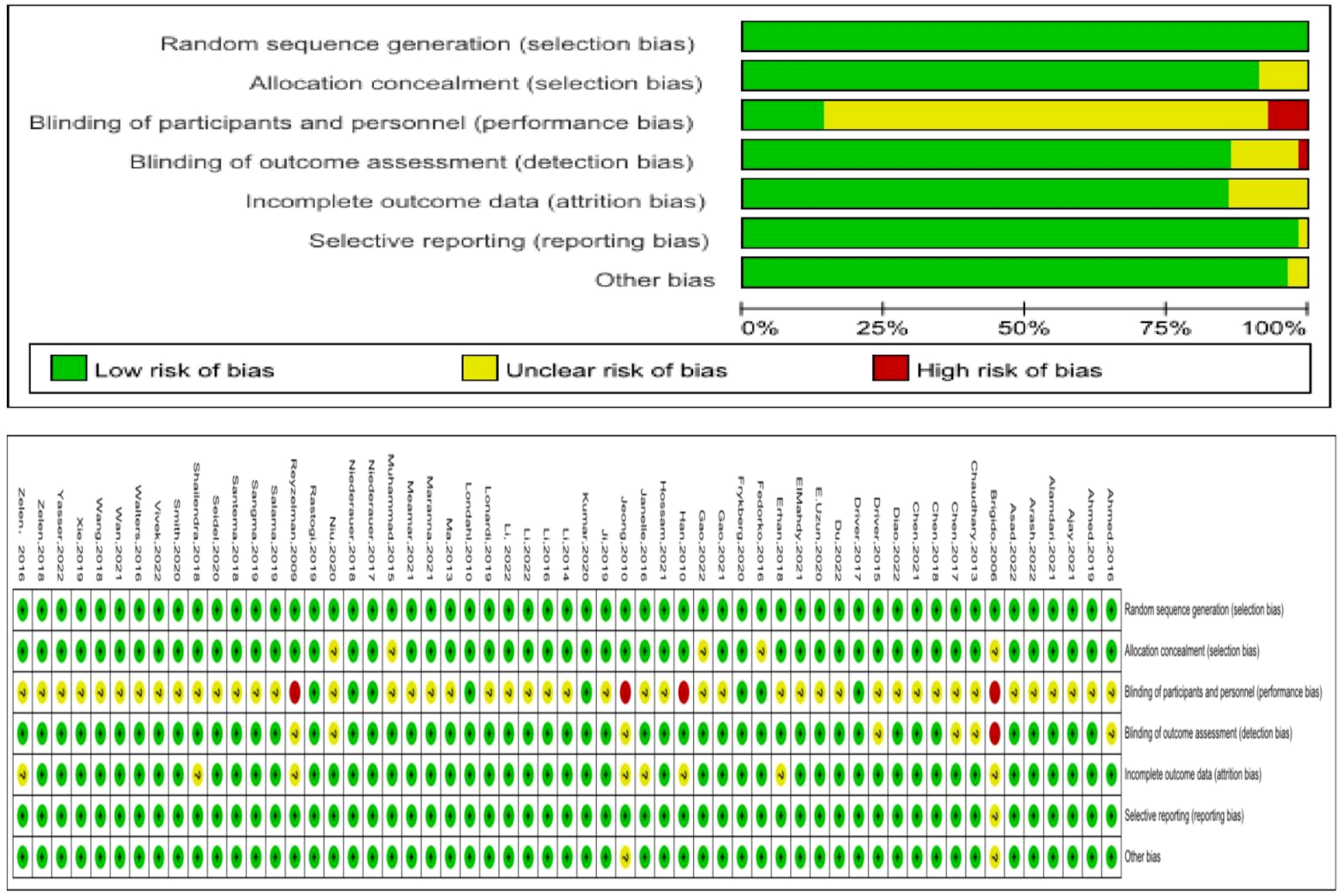
Bias risk assessment of the RCTs.

The quality of the included studies was evaluated, and the results showed that the blinding of the subjects and intervention providers was the main source of potential bias (**Figure 2**). This may be due to the fact that different treatment measures have entirely different approaches, and both patients and researchers are aware of the nature of the study and the allocation of the study group, making blinding impossible.

### Network evidence diagram

**Figure 3:**
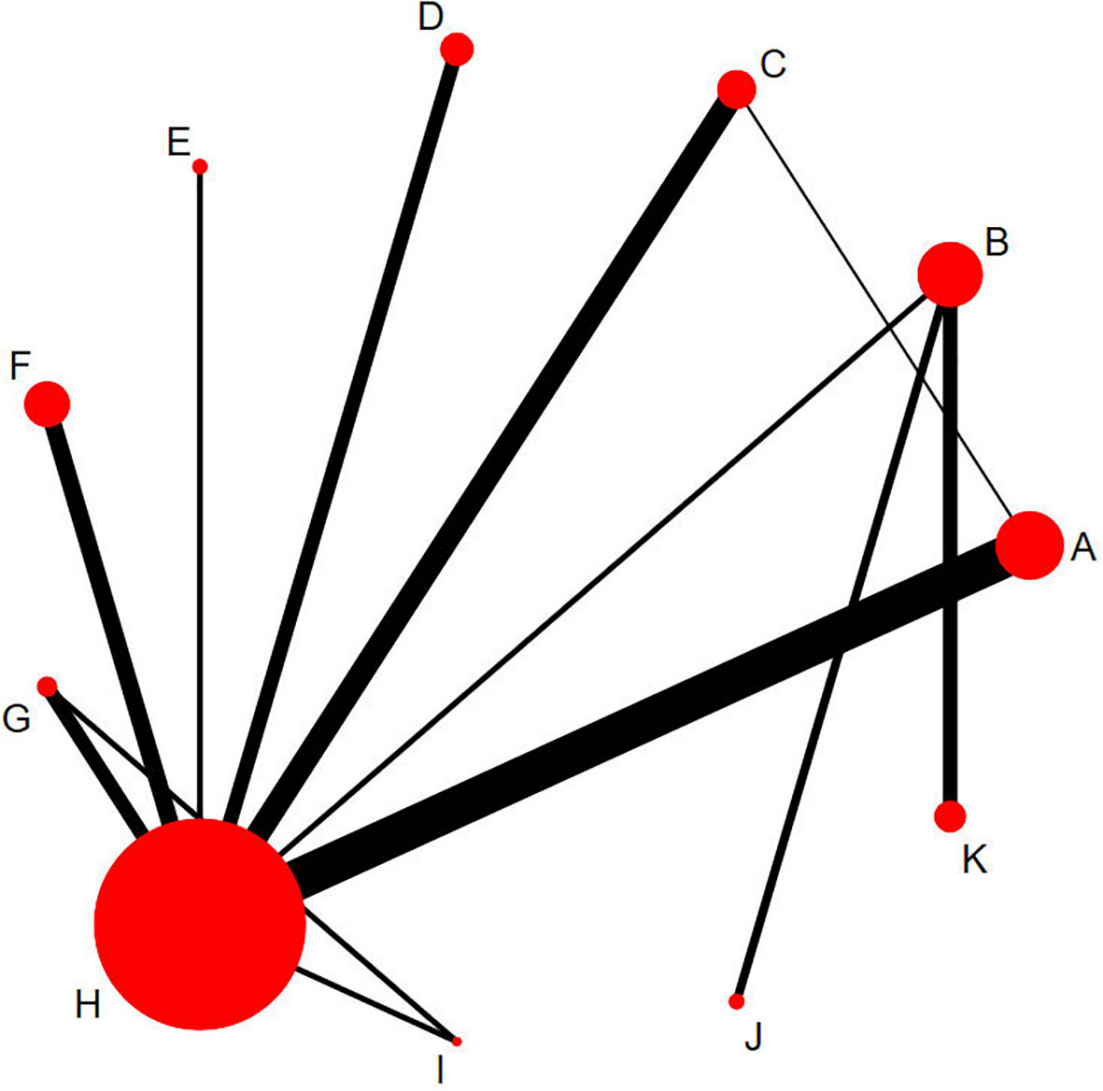
Network plots of eligible comparisons for different treatment strategies. The cirdes represent intervention arms in an RCT-larger cirdes represent presence in more RCTs. The lines connect interventions that were compared in an RCT and thicker connecting lines indicate more direct RCT comparisons. Intervention **(A)**, Platelet-rich plasma; **(B)**, Negative pressure wound therapy; **(C)**, Hyperbaric oxygen therapy; **(D)**, Topical oxygen therapy; **(E)**, Ultrasonic debridement; **(F)**, Acellular dermal matrix; **(G)**, Stem cells; **(H)**, Standard of care; **(I)**, Stem cells+Platelet-rich plasma; **(J)**, Ultrasonic debridement+Negative pressure wound therapy; **(K)**, Platelet-rich plasma+Negative pressure wound therapy.

**Figure 3** shows the mesh map included in the study, and we included the following 11 interventions in the NMA. Each letter represents a different treatment: A=PRP、B=NPWT、C=HBOT、D=TOT、E=UD、F=ADM、G=SCs、H=SOC、I=SCs+PRP、J=UD+NPWT、K=PRP+NPWT.The size of the nodes is proportional to the number of patients, the line between points represents direct comparative evidence, the thickness of the edges is proportional to the number of studies evaluated per intervention, and the edge color represents the average bias risk per head-to-head comparison.

### Results from direct meta-analysis and network meta-analysis

**Table 2** presents the results of DMA and NMA regarding the efficacy outcomes of various therapeutic measures for DFU. Below, the analysis results of different efficacy indicators are described.

**Table 2:** Results of DMA and TMA for diabetic foot outcomes in different treatments measure.

Outcome	Studies (n)	Participants (n)	DMA	NMA
			Effect Estimate (95% CI)	Effect Estimate (95% CI)
Complete wound healing	49	4010		
Standard vs PRP HBOT vs PRP Standard vs NPWT UD+NPWT vs NPWT PRP+NPWT vs NPWT Standard vs HBOT Standard vs TOT Standard vs UD Standard vs ADM Standard vs Scs Scs+PRP vs Scs Scs+PRP vs Standard	14 1 2 3 5 8 5 2 6 5 2 2	1010 40 390 170 439 515 461 160 672 207 34 29	**0.15 (0.069, 0.29)** 0.35 (0.021, 5.7) 0.22 (0.038, 1.1) 2.9 (0.62, 15.0) **3.5 (1.2, 10.0)** **0.37 (0.14, 0.94)** **0.25 (0.078, 0.69)** 0.29 (0.050, 1.7) **0.19 (0.070, 0.50)** **0.12 (0.024, 0.56)** 2.3 (0.23, 23.0) 3.6 (0.033, 42.0)	**0.15(0.070, 0.28)** 0.39 (0.13, 1.2) 0.22 (0.038, 1.1) 2.9 (0.61, 15.0) **3.5 (1.2, 10.0)** **0.37(0.14, 0.92)** **0.25 (0.078, 0.69)** 0.29 (0.050, 1.7) **0.19 (0.070, 0.50)** **0.10 (0.022, 0.40)** 2.2 (0.29, 18.0) **22.0 (2.5, 23.0)**
Reduced ulcer area	19	1626		
Standard vs PRP Standard vs NPWT UD+NPWT vs NPWT PRP+NPWT vs NPWT Standard vs HBOT Standard vs TOT Standard vs UD Standard vs ADM Standard vs Scs Scs+PRP vs Scs Scs+PRP vs Standard	6 3 2 1 1 1 1 1 3 1 1	453 378 223 110 104 73 100 85 90 21 17	-4.0(-9.4, 1.3) -6.2(-14.0, 1.3) 7.1(-2.3, 17.0) 1.1(-12.0, 14.0) -0.11 (-13.0, 13.0) -0.99(-14.0, 12.0) -5.2 (-18, 7.9) 1.1 (-12.0, 14.0) 0.037(-7.7, 7.7) -0.045 (-13.0, 13.0) 4.9(-8.7, 18.0)	-4.0(-9.4, 1.4) -6.2(-14.0, 1.3) 7.1(-2.3, 17.0) 1.1(-12.0, 14.0) -0.094 (-13.0, 13.0) -1.0(-14.0, 12.0) -5.2 (-18, 7.9) (-12.0, 14.0) -0.025(-7.7, 7.6) 2.3 (-9,7, 14.0) 2.4 (-9.7, 14.0)
Time to complete healing	15	1135		
Standard vs PRP Standard vs NPWT UD+NPWT vs NPWT PRP+NPWT vs NPWT	6 2 3 4	413 110 273 339	**3.0(2.2, 3.7)** **3.8(2.6, 4.9)** **-2.1(-3, -1.2)** **-1.3(-2.1, -0.54)**	**3.0(2.2, 3.7)** **3.8(2.6, 4.9)** **-2.1(-3, -1.2)** **-1.3(-2.1, -0.54)**
Amputation rate	17	1706		
Standard vs PRP Standard vs NPWT UD+NPWT vs NPWT PRP+NPWT vs NPWT Standard vs HBOT Standard vs TOT Standard vs Scs	5 3 1 1 6 2 1	408 464 70 110 439 195 20	**6.8(2.2, 41.0)** 1.2(0.41, 3.7) **—** **—** 1.9(0.79, 4.8) 2.5(0.29, 33.0) **—**	**7.0(2.2, 41.0)** 1.2(0.40, 3.8) **—** **—** 1.9(0.79, 4.8) 2.5(0.29, 35.0) **—**
Adverse events	27	2755		
Standard vs PRP Standard vs NPWT UD+NPWT vs NPWT PRP+NPWT vs NPWT Standard vs HBOT Standard vs TOT Standard vs UD Standard vs ADM Standard vs Scs	5 3 3 2 4 3 1 5 1	367 445 170 208 347 341 100 672 105	**3.5 (1.4, 8.9)** 1.4 (0.44, 5.6) **0.098 (0.0082, 0.73)** **0.18 (0.031, 0.90)** 1.1(0.42, 3.0) 1.5 (0.54, 4.2) 1.0 (0.13, 7.8) 1.2 (0.47, 3.0) 3.4 (0.34, 44)	**3.5 (1.4, 8.9)** 1.4 (0.44, 5.7) **0.099 (0.0082, 0.73)** **0.19(0.032, 0.91)** 1.1(0.42, 3.0) 1.5 (0.54, 4.2) 0.99(0.13, 7.9) 1.1(0.46, 3.0) 3.3 (0.34, 43)

The bold values indicate statistical significance.

### Complete wound healing

Forty-nine studies involving 4,010 patients with DFU patients reported on the impact of various interventions on complete wound healing.In both the DMA and NMA groups, we observed that compared to SOC, PRP (0.15, 95% CI, 0.07-0.28), HBOT (0.37, 95% CI, 0.14-0.92), TOT (0.25, 95% CI, 0.078-0.69), ADM (0.19, 95% CI, 0.07-0.5), and Scs (0.10, 95% CI, 0.022-0.40) showed higher ulcer healing rates. When compared to NPWT, the PRP+NPWT group (3.5, 95% CI, 1.20-10.0) had a significantly higher ulcer healing rate. Furthermore, in the NMA, we noted that the complete wound healing in the PRP+stem cell transplantation group (22.0, 95% CI, 2.5-23.0) was significantly higher than that in the SOC. No significant differences were observed among other interventions.

### Reduced ulcer area

Nineteen studies involving 1626 DFU patients reported effects on ulcer area. It was found that in both the DMA and NMA groups, different interventions had no significant advantage in the incidence of reduced ulcer area.

### Time to complete healing

The impact on ulcer healing time was reported in 15 studies involving 1,135 patients with DFU. Research found that in both the DMA and NMA groups, PRP and NPWT effectively reduced the time to complete healing compared to SOC. Moreover, compared to NPWT, UD combined with NPWT and PRP combined with NPWT were more effective.

### Amputation rate

Seventeen studies involving 1,706 patients with DFU reported on the impact of various interventions on the amputation rate. In the DMA, the PRP group had a lower amputation rate compared to the SOC (6.8, 95% CI, 2.2-41.0), with no significant difference observed among other interventions. Similarly, in the NMA group, we also observed a lower amputation rate in the PRP group compared to the SOC (7.0, 95% CI, 2.2-41.0), with no other notable findings.

### Adverse events

Research involving 2755 patients with DFU reported on the impact of different interventions on adverse events. In both the DMA and NMA groups, we observed that compared to SOC, PRP (3.5, 95% CI, 1.4-8.9) resulted in a lower rate of adverse events. When compared to NPWT, the PRP+NPWT group (0.19, 95% CI, 0.032-0.91) and the UD+NPWT group (0.099, 95% CI, 0.0082-0.73) had lower adverse events, while no significant differences were observed among other interventions.

### SUCRA

NMA can evaluate the best effect of each intervention for different results and sort each intervention by SUCRA values, with a higher SUCRA value indicating a better intervention or a lower incidence of adverse reactions. **Table 3** displays the detailed ranking results. Based on **Table 3**, it can be observed that the UD combined with NPWT group is most effective in reducing the ulcer area (91.59%), shortening the ulcer healing time (72.98%), and decreasing the amputation rate (92.15%) and adverse reactions (90.98%). This is followed by the PRP combined with NPWT group. When considering the complete wound healing, the SCs combined with PRP group (83.56%) demonstrates the best efficacy, followed by the PRP combined with NPWT group (80%), and then the UD combined with NPWT group (73.58%). In comparison to other groups, the SOC group is the least effective in promoting ulcer healing (1.52%), reducing the ulcer area (28.06%), shortening the ulcer healing time (0%), and decreasing the amputation rate (12.86%) and adverse reactions (18.45%). Furthermore, it is noted that the treatments of SCs, PRP, and ADM are generally more effective in promoting ulcer healing and reducing amputations compared to NPWT, HBOT, TOT, and UD groups. The efficacy of PRP+NPWT and UD+NPWT in promoting ulcer healing, reducing the ulcer area, shortening the healing time, and decreasing the amputation rate and adverse reactions are superior to that of the alone PRP, NPWT, and UD groups.

**Table 3:** SUCRA Values and Ranks of Efficacy Outcomes.

Interventions	Complete wound healing		Reduced ulcer area		Time to complete healing		Amputation rate		Adverse events	
	SUCRA (%)	Rank	SUCRA (%)	Rank	SUCRA (%)	Rank	SUCRA (%)	Rank	SUCRA (%)	Rank
PRP NPWT HBOT TOT UD ADM SCs SOC SCs+PRP UD+NPWT PRP+NPWT	58.96 40.64 23.99 38.47 34.93 46.91 67.71 1.52 83.56 73.58 80.00	5 7 10 8 9 6 4 11 1 3 2	56.91 67.41 34.21 98.71 59.86 28.71 30.90 28.06 45.83 91.59 67.91	5 3 8 7 4 10 9 11 6 1 2	2.89 22.36 – – – – – 0 – 72.98 51.77	4 3 – – – – – 5 – 1 2	64.24 20.46 36.99 40.65 – – 41.28 12.86 – 92.15 91.37	3 7 6 5 – – 4 8 – 1 2	71.42 36.73 25.98 41.12 27.93 30.10 62.83 18.45 – 90.98 83.47	3 6 8 5 9 7 4 10 – 1 2

### Publication bias

**Figure 4:**
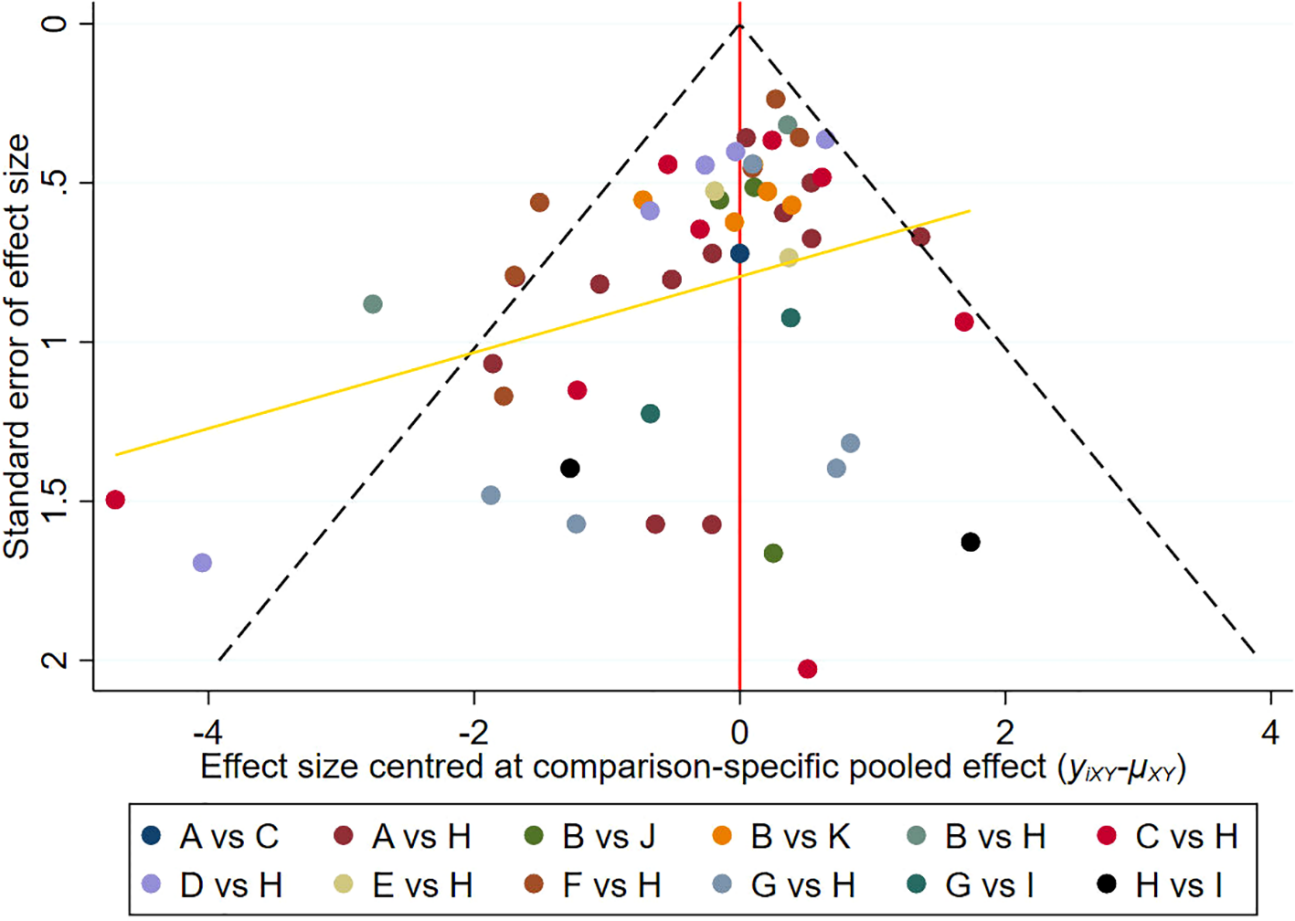
Comparison-adjusted funnel plot. Points of different colors represent different interventions.Each dot represents a direct comparison of different interventions in the study. **(A)**, Platelet-rich plasma; **(B)**, Negative pressure wound therapy; **(C)**, Hyperbaric oxygen therapy; **(D)**, Topical oxygen therapy; **(E)**, Ultrasonic debridement; **(F)**, Acellular dermal matrix; **(G)**, Stem cells; **(H)**, Standard of care; **(I)**, Stem cells+Platelet-rich plasma; **(J)**, Ultrasonic debridement+Negative pressure wound therapy; **(K)**, Platelet-rich plasma+Negative pressure wound therapy.

**Figure 4** displays a comparison-adjusted funnel diagram. Most studies on the funnel map are symmetrically distributed across the vertical lines of X=0, indicating that there were no significant small-sample effects and publication bias.

### Additional analyses

As a *post-hoc* sensitivity analysis, we evaluated the results after removing studies that had scored highly on the Cochrane Risk of Bias Tool. The results showed that after removing the study of Brigido et al., there was no significant difference between the current results and those before the elimination, and the ranking order remained consistent, which proved that the results of network meta-analysis were reliable.

## Discussions

DFU form the basis for 40 - 70% of non-traumatic lower extremity amputations in DM, often leading to disability and severely impeding the quality of life (88, 89). The Wound Healing Society (WHS) pointed out in its 2013 DFU treatment guidelines that “promoting decompression, reducing bacterial and cellular burden through adequate debridement, while using moist dressings for local wound care, absorbing wound exudate, and local and systemic antibiotic treatment when necessary, as well as treating osteomyelitis.” Moreover, the guidelines also stated that cell and non-cell equivalents improve DFU healing by releasing growth factors, cytokines, and proteins that stimulate the wound bed (5). Therefore, besides the first-line conventional treatments, in recent years, several new treatment modalities such as NPWT, TOT, HBOT, growth factors, bioengineered skin substitutes, and electrophysical therapy have brought new opportunities to DFU patients, yet consensus has not been reached (18). Numerous studies have currently been conducted to compare these treatments and analyze their impact on DFU outcomes, thereby evaluating the efficacy and safety of these drugs. However, DMA mainly focuses on the effects of two different interventions, and few studies have compared the effects of multiple different treatments. Therefore, we have collected relevant published RCTs and used NMA to directly and indirectly compare the effects of different treatments on DFU outcomes, including complete healing rate, ulcer area reduction, time required for ulcer healing, amputation rate, and adverse reactions. Our aim is to provide clinicians with information on the risks and benefits of different treatment options when choosing different DFU treatment strategies.

Normal wound healing is a meticulously orchestrated process, involving a series of complex and continuous interactions between regulatory cytokines. The four main stages of wound healing are the coagulation phase, inflammation phase, proliferation phase, re-epithelialization, and remodeling phase, which result in the restoration of tissue functional integrity (90, 91). Most wounds heal within 2-4 weeks. If the healing process is interrupted and the skin structure and functional integrity cannot be restored within 3 months, it is usually referred to as a chronic or refractory ulcer (92). However, due to changes in the microenvironment caused by DM, including changes in oxygen levels, chemokines, growth factor synthesis, extracellular matrix, and oxidative stress, which alter normal cell recruitment and activation, and lead to impaired or delayed wound healing (89). Such wounds deviate from the normal process, remain unhealed for long periods, forming chronic refractory ulcers in DM, making the treatment riskier, more time-consuming, and more expensive. Currently, it is widely believed that peripheral nerve lesions and peripheral vascular lesions are the two main factors causing DFU in patients with DM (93). Secondly, the high glucose environment and inflammation disorder in diabetes patients are also major factors leading to the difficulty in healing DFU (94–96). Therefore, how to promote the healing of DFU has become a challenge.

Traditional wound care employs various enhanced dressings to promote wound healing, yet these dressings often adhere to the wound scab, altering the process and damaging new granulation tissue, thereby impeding wound healing (97). NPWT is a state-of-the-art noninvasive adjunct therapy system. It leverages VSD or VAC devices to maintain negative pressure. By connecting to a sealed dressing and tube from an open wound to a collection vessel, it removes fluid, thereby facilitating wound healing (98–100). Previous meta-analyses have indicated that NPWT treatment for DFU has a higher ulcer healing rate, shorter healing time, and lower amputation rate compared to SOC (101, 102). In a meta-analysis by Lin et al., it was observed that the healing rate in the NPWT group was significantly higher than in the SOC, while the granulation tissue formation time was significantly shorter. However, there was no statistically significant difference in the incidence of adverse events or amputations between the two groups (21). This aligns with our findings, where the results from DMA and NMA indicate that NPWT does not offer superiority in terms of complete ulcer healing or reduction in ulcer area compared to SOC. However, it does reduce the time required for ulcer healing without increasing the incidence of adverse reactions or amputations. The SUCRA ranking suggests that NPWT is superior to SOC in all aspects of the study. In addition, UD is also a non-contact wound treatment method. Both Singh and Tehrani have found that the use of UD in patients with DFU can significantly accelerate the healing process (103, 104), although its long-term complete healing response seems less favorable. In the latest meta-analysis by Chen and others, it was found that compared to SOC, the use of UD significantly increases the healing rate of wound ulcers and results in a higher percentage reduction in wound ulcers (20). In our study, it was observed that compared to SOC, the UD group showed no significant advantages in complete healing rate or reduction in ulcer area, possibly due to the insufficient number of included studies. However, in terms of adverse reactions, UD is as safe as standard treatment. Ruran and others conducted a meta-analysis comparing NPWT and UD in the treatment of DFU, and found that NPWT is similar to UD in treating DFU, but surpasses standard wound care in terms of efficacy and safety (105). In our study, we further analyzed the efficacy of UD combined with NPWT in treating DFU. The study found that compared to alone NPWT treatment, UD combined with NPWT treatment shows similar efficacy in terms of complete healing rate and reduction in ulcer area, but has an advantage in terms of shortening the healing time of ulcers and reducing adverse reactions. The SUCRA ranking results suggest that UD combined with NPWT is more effective than alone UD, NPWT, or SOC in terms of complete healing rate, reduction in ulcer area, and shortening the healing time of ulcers, while also having lower adverse reaction and amputation rates. In diabetic patients, under the state of elevated blood glucose, the peripheral nerves and blood vessels are subjected to abnormal cell proliferation, vascular endothelial cell disorder, the micro-environment change, and inflammatory response, which are the main reasons why DFU is difficult to heal (106). By using different internal and external pressures, NPWT can drain deep necrotic tissue and secretions, reduce wound infection, keep the wound moist, and promote wound healing (107). The mechanisms of NPWT at the tissue and cellular level include: promoting granulation and angiogenesis, wound boundary epithelialization, and promoting cell migration and proliferation. The large strain mechanism includes wound edge closure and clearance of infectious material exudate (108). UD can remove bacteria, fungi, and necrotic tissue through the effects of cavitation and hemostasis during high-frequency and high-energy ultrasonic jet irrigation. Compared to traditional debridement techniques, UD not only helps to control infections but also promotes ulcer surface healing through thermal and biological effects. The thermal effect is manifested by increased skin temperature and improved blood supply, which facilitates tissue repair (109), while the biological effect is observed in low-frequency ultrasonic waves that indirectly promote the release of growth factors, accelerating ulcer healing more quickly (110). Therefore, when the two are used in combination, it can not only improve the wound microenvironment, change the microvascular hemodynamics, control the wound infection, but also promote the regeneration of endothelial cells and promote faster healing of ulcers. There was no increased incidence of adverse effects, and amputation rates were lower than with conventional treatment.

In addition to the combined treatment with UD, NPWT can also be used in conjunction with PRP. PRP is a preparation rich in platelets, with a concentration higher than that of whole blood, and its platelet concentration in the plasma is 4-5 times higher than that of whole blood (111, 112). PRP can be applied alone to DFU or in combination with other treatments. In the two recent meta-analyses, Gong and Peng et al. found that PRP treatment for DFU increased the likelihood of wound healing, reduced the ulcer volume, and decreased the time required for complete wound healing (25, 26). Tasmania et al.’s meta-analysis also showed that in terms of safety, there was no difference or recurrence in the probability of wound complications between PRP and SOC, but it significantly reduced the incidence of adverse events overall (27). In addition, multiple studies have found that PRP combined with fat-derived stem cell transplantation can promote angiogenesis and increase transplantation rates (113, 114). Yin et al.’s meta-analysis showed that compared to the control group, NPWT combined with PRP had significant advantages in terms of reducing healing time, improving ulcer healing rates, and shortening hospitalization duration, but there was no significant difference in dressing change time and hospitalization costs (22). However, whether PRP treatment for DFU results in later amputation and the extent of amputation is still unclear. Our study found that both DMA and NMA results showed that when applied alone, PRP could improve the complete healing rate, reduce healing time, and decrease the amputation rate and adverse reaction rate compared to the standard treatment group, but there was no significant difference in reducing the ulcer area. Our study also found that when combined with NPWT treatment, PRP could effectively improve the complete healing rate, reduce healing time, and decrease adverse reactions compared to NPWT treatment alone, but there was no significant difference in reducing the ulcer area. The main mechanism of PRP in wound healing is through the release of various bioactive molecules stored in platelets, including PDGF, transforming growth factor β(TGF-β), VEGF, epithelial growth factor (EGF), and adhesion molecules such as fibrin, fibronectin, and hyalenin (115). These factors are known to regulate cell migration, adhesion, proliferation, and differentiation, and promote the accumulation of extracellular matrix (ECM) by binding to specific cell surface receptors, thereby playing an important role in wound healing and regeneration (115, 116). In addition to growth factors, PRP include many important proteins, such as fibrin and antibacterial proteins, which not only provides scaffolds for tissue regeneration, promotes wound contraction, blood clotting and wound closure, but also inhibits bacterial growth (117–119). Furthermore, our study revealed that compared to standard treatment, when combined with SCs, PRP had no significant statistical difference in complete healing rate as shown in DMA results, but in NMA, it showed a higher complete healing rate.

In chronic wounds, the affected tissues are oxygen-deficient, impeding the healing of ulcers. An increase in oxygen levels in wound tissues often indicates better wound healing and fewer bacterial colonizations (120). Therefore, oxygen plays a significant role in the healing of chronic wounds. HBOT involves breathing 100% oxygen at pressures two to three times higher than normal atmospheric pressure in a hyperbaric chamber, leading to an increase in oxygen tension in both arteries and tissues. It can improve local tissue oxygenation, and further research suggests that HBOT may improve new blood vessel formation, stimulate stem cells and growth factors, inhibit inflammation, and have antibacterial effects on anaerobic bacteria (121). There are different views on the effectiveness and safety of HBOT in DFU. Research by Zhao and colleagues showed no differences in terms of ulcer incidence, amputation risk, or adverse events compared to SOC (30). However, a study by Sharma and colleagues believed that HBOT had a significant effect on the complete healing of DFU, while also reducing healing time and the risk of major amputations. Additionally, this study found no differences in the reduction of ulcer area or average percentages of mortality, and the SOC had fewer adverse events (24). In our study, we found that both HBOT and TOT can improve the complete healing rate of ulcers, but there were no differences in terms of reducing the area of ulcers, amputation rates, or adverse events. The SUCRA ranking results suggest that both HBOT and TOT treatments are superior to SOC in all aspects of the study.

In addition to considering the efficacy and safety of the treatment, when we choose the treatment for diabetic foot patients in the clinic, we also need to further consider the economic benefits of the treatment for patients and whether ethical support is needed. The hospitalization cost of DFU increases with the severity of the disease. It generally depends on the extent of the ulcers and the underlying pathology that caused them in the first place, but also on the interventions used to treat them. Therefore, at the initial visit, it is important that the clinician adequately assess the patient for potential complications as well as the wound itself, and determine whether peripheral artery disease, neuropathy, or both are present. Therefore, cost-effective measures are necessary to reduce intervention costs in treating DFUs and thus decrease the economic burden associated with them (122, 123). When it comes to the use of stem cells to treat diabetic foot patients, we also need to obtain the relevant ethical approval and informed consent of the patient. In addition, DFUs affect multiple areas of a person’s functioning, including both physical and psychological distress. Therefore, it is important to pay attention to the psychosomatic lectures of diabetic patients.

Our study provides a comprehensive analysis of the current primary treatments for DFU, incorporating the latest RCTs and ranking various treatment outcomes. This ensures our results are detailed and robust, offering clinicians a reliable foundation for choosing DFU treatment options. However, our study has its limitations. Firstly, our research population varies in terms of race, background, and age, and there are fewer studies on certain observed indicators and treatment measures. This warrants a future RCTs involving a broader range of regions and populations for further analysis. Secondly, the measurement and timing errors in the observed indicators included in our study may vary across different studies, which could potentially affect clinical efficacy. Lastly, since different treatment approaches have distinct pathways, it is impossible to prevent blindness. This is the main reason for the potential biases in our study. Nevertheless, the conclusions and limitations of this study may offer some guidance for the design of new trials.

## Conclusion

The treatment options for DFU are not singular. Research has found that combining multiple methods often yields better outcomes without increased adverse reactions. Therefore, when confronting patients with DFU, clinicians can choose one or multiple methods based on the actual condition of the ulcer, and evaluate the efficacy and risks of different treatment plans according to various scenarios. Future research requires more clinical trials to investigate the effectiveness of combined treatments for DFU.
